# Utilisation of quality antenatal, delivery and postnatal care services in Nepal: An analysis of Service Provision Assessment

**DOI:** 10.1186/s12992-021-00752-x

**Published:** 2021-09-06

**Authors:** Resham B. Khatri, Jo Durham, Yibeltal Assefa

**Affiliations:** 1 School of Public Health, Faculty of Medicine, University of Queensland, Brisbane, Australia; 2 Health Social Science and Development Research Institute, Kathmandu, Nepal; 3 School of Public Health and Social Work, Queensland University of Technology, Brisbane, Australia

**Keywords:** Technical quality, Nepal, antenatal care, delivery and postnatal care services, health facility, utilisation

## Abstract

**Background:**

Nepal has improved access and utilisation of routine maternal and newborn health (MNH) services. Despite improved access to routine MNH services such as antenatal care (ANC), and delivery and postnatal care (PNC) services, the burden of maternal and neonatal deaths in Nepal remains high. Most of those deaths could be prevented by improving utilisation of evidence-informed clinical MNH interventions. However, little is known on determinants of utilisation of such clinical MNH interventions in health facilities (HFs). This study investigated the determinants of utilisation of technical quality MNH services in Nepal.

**Methods:**

This study used data from the 2015 Nepal Services Provision Assessment. A total of 523 pregnant and 309 postpartum women were included for the analysis of utilisation of technical quality of ANC, and delivery and PNC services, respectively. Outcome variables were utilisation of better quality i) ANC services, and ii) delivery and PNC services while independent variables included features of HFs and health workers, and demographic characteristics of pregnant and postpartum women. Binomial logistic regression was conducted to identify the determinants associated with utilisation of quality MNH services. The odds ratio with 95% confidence interval (CIs) were reported at the significance level of *p* < 0.05 (two-tailed).

**Results:**

Women utilised quality ANC services if they attended facilities with better HF capacity (aOR = 2.12;95% CI: 1.03, 4.35). Women utilised better quality delivery and PNC services from private HFs compared to public HFs (aOR = 2.63; 95% CI: 1.14, 6.08). Women utilised better technical quality ANC provided by nursing staff compared to physicians (adjusted odds ratio (aOR) =2.89; 95% CI: 1.33, 6.29), and from staff supervised by a higher authority compared to those not supervised (aOR = 1.71; 95% CI: 1.01, 2.92). However, compared to province one, women utilised poor quality delivery and PNC services from HFs in province two (aOR = 0.15; 95% CI: 0.03, 0.63).

**Conclusions:**

Women utilised quality MNH services at facilities with better HF capacity, service provided by nursing staff, and attended at supervised HFs/health workers. Provincial and municipal governments require strengthening HF capacities (e.g., supply equipment, medicines, supplies), recruiting trained nurse-midwives, and supervising health workers.

**Supplementary Information:**

The online version contains supplementary material available at 10.1186/s12992-021-00752-x.

## Introduction

Over the past two decades, Nepal has made significant improvements in accessing routine maternal and newborn health (MNH) services such as antenatal care (ANC), delivery and postnatal care (PNC) services [1]. Evidence indicates improvement is associated with the implementation of the Safe Delivery Incentive Program (SDIP) (called as Aama Program in Nepali) in 2006 in Nepal [2], a nationwide program providing conditional cash transfers to women who deliver at a health facility (HF) assisted by skilled birth attendants (SBAs) and who attend at least four ANC visits and delivery, mostly in public HFs and some private HFs [2, 3]. Nevertheless, reducing the neonatal and maternal mortality ratio (MMR) remains challenging, e.g., from 2006 to 2016, the institutional delivery rate increased from 18 to 59%, while MMR decreased only from 281 to 259 (reported as per 100,000 live births) in that period [1]. A study in India also showed that improved access to MNH services has not significantly reduced maternal and neonatal deaths [4]. One explanation for this is that the quality of care that women receive across the MNH continuum of care (CoC) is poor and therefore is having a limited effect on mortality.

The quality of health care is multifaceted, and its measurement is complex. According to the Donabedian’s model of health care, quality of health care is comprised of three components: input (or structural quality), process, and outcome [5]. Structural or input quality (HF capacity) or health system readiness is the precondition for better quality technical/clinical care. Process of care is the delivery of health services, and it is often seen as consist of two components: technical quality, and social, or perceived quality [6]. Outcome quality is an effect of health care and is generally measured in terms of improved health status or reduced morbidities or mortalities. Thus, the measurement of health care quality is the assessment of health system quality, and considers overall health system inputs, delivery and utilisation of care, and effects of health care, including client satisfaction and respectful and dignified care [7, 8]. Health system quality means better health system readiness, delivery and utilisation of quality health services by those who need them [7].

The clinical or technical quality of MNH services is measured by the delivery of sets of clinical interventions and procedures during services delivery by trained health providers or received by health service users [9, 10]. The health care utilisation (probability receiving health interventions from the available providers at HFs) incorporates access to health services (reaching the care) as well as care provided in the HFs by providers (provision of care) [11]. The utilisation of better technical quality of MNH services by mothers and newborns can have a real effect on the reduction of maternal and neonatal morbidities and mortalities [9, 12]. In addition to technical quality, the client’s perception of quality is important and is dependent on the sociocultural context and individual perceptions [13]. However, perceived quality can also be affected by the social desirability bias (reporting better/positive behaviours from fear of not being bad) [14].

Studies conducted in Nepal have mainly focused on uptake of specific MNH interventions from household survey data [15–17] while further analysis of facility survey data have mainly looked at access to health services [18] or HF capacity (structural quality) or health system readiness [19, 20] or perceived quality of MNH care [13, 21]. A study by Karkee and colleagues reported that public hospitals and peripheral HFs were rated lowest if HFs had inadequate medical equipment and room, lack of privacy, inadequacy of health staff trained on women’s health, lack of provision of adequate water supply, clean environment and privacy [22]. In another study, women perceived better quality of care in private HFs [23]. The same study reported women also bypassed peripheral HFs, preferring to attend referral hospitals for perceived better quality maternity care. A study by Khatri and colleagues revealed women preferred home delivery without skilled providers due to poor perceived quality of care at local HFs [24]. Other research also indicated HFs in province two demonstrated poor health system readiness for non-communicable diseases (NCDs) compared to province one [25], and inadequately staffed HFs for the delivery of MNH services [26].

Studies in Nepal, however, are lacking on the determinants of utilisation of better clinical/technical quality MNH services at HFs. Current routine health information system lacks data to measure the quality of MNH care and track the determinants of utilisation by pregnant and postpartum women. The 2015 Nepal Service Provision Survey (SPA) collected information on utilisation of health service using HF inventory, observation of interactions of health services providers and clients, and client exit interviews of health services users on the day of the HF survey. The HF inventory is the process of collecting information on the availability of equipment, medicines, health workforce and availability of protocols/guidelines of services, observation and review of records, and interviews with those in-charge of HFs. Further analysis of the data of SPA 2015 could provide potential indicators to track policy implementation and patterns of utilisation of quality MNH services. Therefore, this study aimed to examine the determinants of utilisation of better technical quality MNH services in Nepal. The study’s findings can inform decision-makers in the revision of programmes and strategies for achieving universal coverage of quality MNH services and Sustainable Development Goal three (SDG3).

### Nepal’s health system and policy context for MNH

**Fig. 1:**
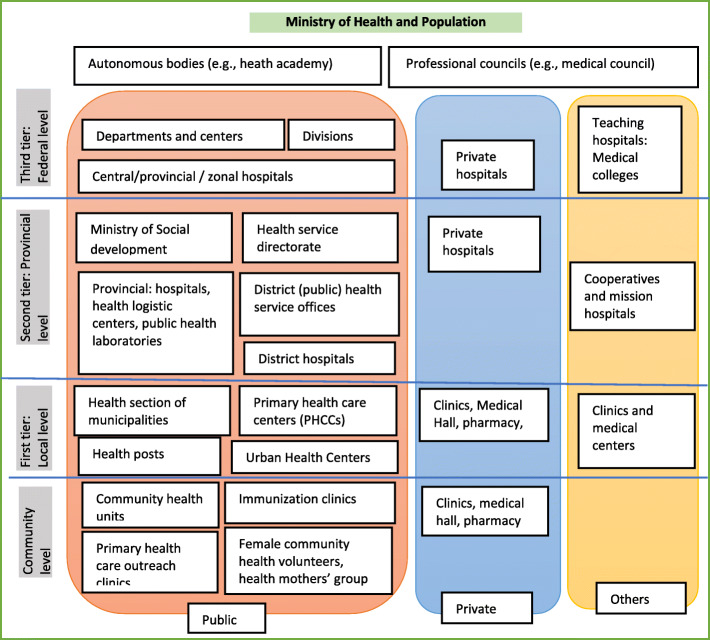
Schematic diagram of health system organogram of Nepal. Source: Prepared by the first author (RBK) based on information from the Department of Health Services (dohs.gov.np); Annual Health Report 2018 [27], and previous studies [28, 29]

Nepal has a three-tier federal health system in line with the governance system: federal, provincial, and local governments (municipal governments) (Fig. 1). The current health system has decentralised the resources and authority to provincial and local/municipal governments [30]. The federal republic constitution of Nepal (2015) recognises basic health services as fundamental rights, and available through public funding [31].

The health care delivery system is mixed with private providers dominant in tertiary and secondary health care services and concentrated in urban areas in Nepal [32]. Province three, including the federal capital (Kathmandu), has the highest private HFs [33]. The public health system provides basic health services and secondary health care services in urban, rural and regional areas through public funding. People require to pay health care beyond basic health services in both public and private HFs [34]. The proportion of out of pocket (OOP) expenditure is high (57% of current health expenditure) [35]. In private HFs, people must pay for basic health services, including routine MNH services [33]. The cost of care of those services is low in public facilities but expensive in private HFs. Since 2016, Nepal has implemented the National Health Insurance Program (NHIP), primarily focusing on point of care public facilities and referral to contracted private HFs; however, private HFs have very limited participation in the program, low enrollment rate and high dropout rate in the renewal of premium further challenge the implementation of the NHIP [36].

Under the publicly funded National Safe Motherhood Program, MNH services are delivered up to the local level HFs free of cost [27]. Institutional delivery service is available from the health post level with skilled birth attendants (such health posts are also called birthing centers) to tertiary level facilities. Two important policy shifts in 2005 were the Skilled Birth Attendant (SBA) policy and the Safe Delivery Incentive Policy (SDIP) [37]. The SBA policy shifted the task of childbirth services to SBAs (e.g., auxiliary nurse midwives with two-months long in-service midwifery training) at the birthing center level, while to address the demand side financial barriers, the SDIP provisions a minimal monetary incentive (≈10 USD) for women who complete 4ANC visits and gave birth in accredited HFs [38]. The SDIP or “Aama” program is implemented in most public facilities and a limited number of private HFs [32]. More recent policies [39–41] and the Nepal Health Sector Strategy (NHSS) 2016–2021 [42] have focused not only on demand but also improved quality MNH services and to contribute to the SDG3 [41, 43]. Nepal’s Road to Safe Motherhood Plan 2030 [44] also emphasises universal coverage of quality MNH services to achieve the SDG3 [45].

## Methods

### Study context

Nepal is ethnolinguistically diverse country with a population of 29 million, residing125 different caste/ethnic groups who speak 123 languages (Nepali is the official language) [37]. Life expectancy is 70.8 years (male: 69.3 and female: 72.2), 60% are living in urban areas [46]. More than one in three (34%) people live below the poverty level based on the multidimensional poverty index [47]. Additionally, two-thirds of Nepal’s gross national product (GNP: USD 30.64B) is from agricultural sector (37%) and remittance from foreign employment (32%) [48].

**Fig. 2:**
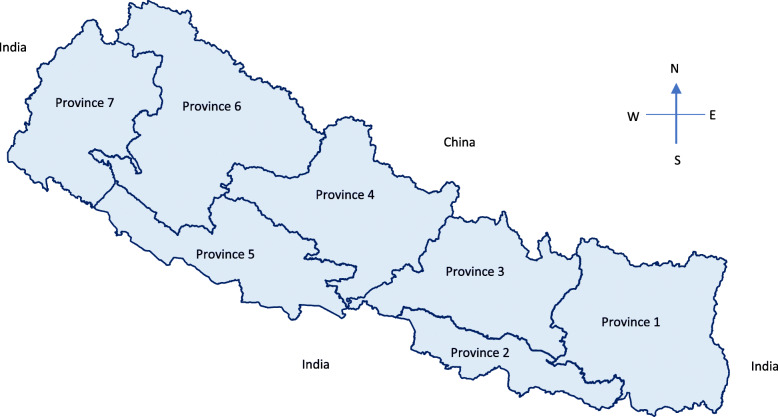
The provincial map of Nepal. Source: Map is prepared in word processer document. The shape files were obtained from the Government of Nepal, Ministry of Federal Affairs and Local Development and were publicly available for unrestricted use (https://data.humdata.org/dataset/admin-shapefiles-of-nepal-mofald)

As presented above, the health system has three tiers, and service delivery is provided by a mix of public and private HFs. There are 6.7 health workers per 10,000 populations, which is significantly less than the World Health Organization (WHO) standards (23/10,000 population) [34]. Of the seven provinces in Nepal (Fig. 2), province six is the most remote province where HFs are scattered and often difficult to reach, especially those in more rural/remote areas of the province. Compared to other provinces, province two is geographically accessible, but has low socioeconomic indicators and the highest number of childbirths annually [32].

### Study design

This was a cross-sectional study based on further analysis of the data derived from the 2015 Nepal SPA (also called as the 2015 Nepal Health Facility Survey) [29]. The 2015 Nepal SPA was a cross-sectional study representative of the main types of HFs in Nepal, was based on generic designs with modules developed by the Measure DHS, ICF Macro. The SPA tools were revised and modified to Nepal’s context and aligned with WHO’s Service Availability and Readiness Assessment (SARA) [49].

### Sampling design and data collection

In the 2015 Nepal SPA, data were collected using the Donabedian model of quality of health care measurement (inputs-process-outcome) [29]. Inputs related information were collected items derived from the SARA Manual [49]. All input related information were collected using the HF inventory (process of collecting information through observations of stores, reviewing records, and interactions with the most knowledgeable person in HFs, usually HF in charge) (questionnaire pages 225–332 of the 2015 Nepal SPA original report) [29]. Information on different services specific processes of care (utilisation of services) and outcome (e.g., client satisfaction) were collected by observation of interactions of health services and clients and exit interview on the day of HF survey (questionnaire pages 333–415 of the original report) [29].

The 2015 Nepal SPA was a two-stage cluster survey where 992 HFs(963 HFs when weighted) were selected randomly from the master list of 4719 (HFs proportionate representation of three regions and types of HFs). In addition, interviews with health workers were conducted. In total, the survey included 523 pregnant women who received the first ANC visit (minimum one to maximum four pregnant women per HF, selected from 269 HFs) and 309 (minimum one to four postpartum women per HF, selected from 109 HFs) postpartum women who received delivery and PNC services at the HFs on the day of the survey were included in the analysis. In the current study, multiple sources of data were used: HF inventory, observation of health services delivery, and client exit interviews with pregnant women who attended for first ANC visit, and postpartum mothers who discharged on the day of survey. Multiple data files (HF inventory, interview with health workers, observation of interactions of women-providers, and client exit interviews) for each outcome variable were merged into one file using a unique identifier (HF number) available in the data file. The unit of analysis was pregnant women for ANC services, and postpartum women for delivery and PNC services.

### Conceptual framework of the study

**Fig. 3:**
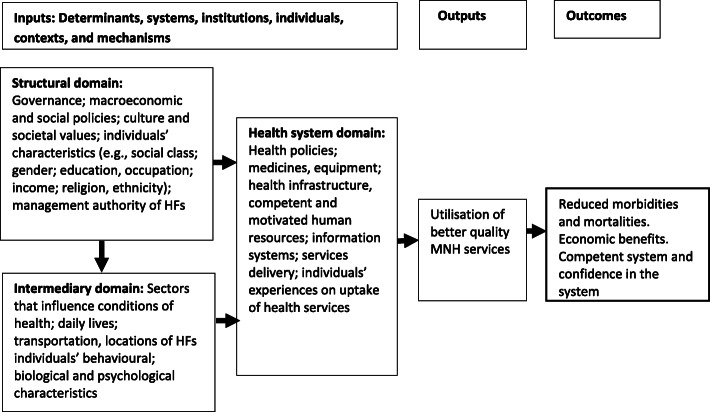
A conceptual framework to guide the analysis of this study

Based on the review of previous conceptual frameworks [50–52], including WHO’s SDH framework [52], a conceptual framework was developed (Fig. 3).

The conceptual framework included inputs that consisted of several determinants, their contexts and mechanisms which may act at different levels (system, organisation and individual) [53]. These contexts and mechanisms produce the outputs of utilisation of quality MNH services. Utilisation of better quality MNH services leads to the survival of mothers and newborns, economic benefit, and confidence in the system [7]. Inputs include determinants into structural, intermediary and health system domains. The structural domain covers all basic sociopolitical system and organisational factors and individual structural determinants(e.g., governance, wealth status, ethnicity, gender) [52]. The intermediary domain covers non-health sector underlying factors that affect the conditions of health via influencing the family/community contexts (living and working conditions), and characteristics of individuals such as biological, behavioural and psychological determinants [54]. Structural factors influence the non-health sector system and organisational factors, and characteristics of individuals. The health system domain includes several variables that can influence the provision and delivery of quality health services, including MNH services [55]. Determinants of structural and intermediary domains influence the systems, organisational and characteristics of health services users of health system domain.

### Study variables

In this study, the unit of the analysis was health service users (pregnant women and postpartum women). Based on the information available in the 2015 Nepal SPA, independent variables included characteristics of women, characteristics of health workers from where they received MNH services, and the HFs women attended (See details in supplementary file Table S1). Based on previous literature [52, 53], these variables were grouped into three domains as guided by the conceptual framework (Fig. 3). Structural variables included women’s ethnicity, the managing authority of HFs (public, private), and the education level of women. Intermediary variables included province, region, age of women, facility type, companion at HFs (usually, husband, mother-in-law), and waiting time. Health system variables included HF capacity, types of HWs, mode of delivery, several health management-related variables (Supplementary file Table S1). The HF capacity (for ANC services, and delivery and PNC services) was also considered an independent variable and calculated using Principal Component Analysis (PCA). The PCA generates composite-scale coefficients that can be used to reduce indicators to indices for application in comparative analyses of service readiness [56]. To calculate the HF capacity, a total of 53 items were taken for ANC services (Supplementary file Table S2), and 73 items were taken for delivery and PNC services (Supplementary file Table S3). All these items were then categorised into yes/no and later converted into dummy variables by assigning the value of 1 to ‘Yes’ and 0 to ‘No’. Taking an arbitrary cut-off point of HF index based on previous studies [57, 58], HF capacity was categorised into three groups: low, medium and high.

### Outcome measurement

Two outcomes of this study were utilisation of technical quality, i) ANC services, and ii) delivery and PNC services. The WHO recommends every pregnant woman should receive recommended interventions for healthy pregnancy and childbirth. The guideline of the Nepal Safe Motherhood Program also defines the list of MNH interventions to be taken by every pregnant, postpartum woman and newborn [59]. The 2015 Nepal SPA also collected information on ANC (Supplementary file, Table S4) and delivery and PNC interventions (Supplementary file, Table S5) used to assess the technical/clinical quality of MNH services. All ANC, delivery and postnatal interventions were denoted as yes or no categories, whether they received those interventions or not in the HFs visit. Each item had a dummy value of ‘yes’ (=1) if items were available on the day of the survey; otherwise, ‘no’ (=0) (for instance, if iron tablets available in HFs (=1), if not (=0)). The PCA procedure was conducted separately to calculate the quality of ANC services, as well as delivery and PNC services. Using PCA procedure, the quality score of each outcome variable was dichotomised. The PCA procedure takes the arbitrary cut-off point to dichotomise the technical quality score [57, 58]. The utilisation of technical quality of ANC services was categorised: Poor quality =0; better quality =1. The second outcome variable, i.e., delivery and PNC services was also dichotomised using a similar PCA procedure.

### Data analysis

Descriptive and logistic regression analyses were conducted. Descriptive statistics were reported as frequencies and proportions (%). Binomial logistic regression analysis was conducted to identify the determinants of utilisation of better technical quality for each outcome variable. First, bivariable logistic regression analysis was conducted to identify the crude estimates of the association of each independent and outcome variable of interest. Before running the multivariable regression models, multicollinearity was checked and excluded independent variables having variation inflation factor ≥ 3 [60]. In the multivariable analysis, backward stepwise elimination technique was conducted. In each step of the backward elimination logistic regression process, the least significant variable at each step (which has the highest *p*-value in the model) was excluded and repeated the regression analysis. This process was repeated until the stopping rule was satisfied when all remaining variables in the model have a p-value smaller than pre-specified threshold (*p* < 0.20) [61, 62]. In the final regression model, the statistical significance level was *p* < 0.05 (two-tailed). Again, to confirm the final regression model, we run the backward stepwise elimination logistic regression model a) entering only potential risk factors with *p* < 0.20 obtained in the bivariate analysis for backward elimination process, and b) testing the backward elimination method by including all potential risk factors. The goodness of fit test was conducted using the Hosmer Lemeshow test (non-significant results at *p* > 0.05 indicated an adequate fit). Regression analyses outputs were reported as the Odds Ratios (ORs) with 95% confidence intervals (CIs). The clustering effect was adjusted using sampling design in the data analysis stage using the clients’ weight and accounting for survey strata: region and types of HFs. All analyses were conducted using the “svy” command function and considering the clustering effect in Stata 14.0 (Stata Corp, 2015).

## Results

### Descriptive characteristics of women (pregnant and postpartum)

Table 1 shows the descriptive characteristics of women who attended HFs for the first ANC visit, and women who delivered at HFs and discharged on the day of the facility survey. Among the 523 pregnant women who made the first ANC visit, three in five (58%) received services from primary health care centres (PHCCs) and hospital HFs. The majority (86%) of pregnant women received ANC services from publicly managed HFs. More than two in five (44%) pregnant women were aged 20–24 years, and nearly one-third (30%) of pregnant women had never been to school. Among 309 postpartum women, more than two-thirds (69%) received delivery and PNC services from public facilities. Half (50%) were from privileged (Brahmin/ Chhetri) ethnic groups. One-third (34%) of women were from province three. Nearly four in five women (77%) had normal deliveries. Two-thirds of women (66%) utilised delivery and PNC services from midwives and nurses.

**Table 1:** Descriptive characteristics of women who received routine MNH (ANC visit, and delivery and PNC services) services, Nepal SPA 2015

Pregnant women attended HFs for their first ANC visit (***N*** = 523)				Postpartum women discharged from HFs (***N*** = 309)			
**Determinants**	**Categories**	**Frequency**	**%**	**Determinants**	**Categories**	**Frequency**	**%**
**Structural**				**Structural**			
Ethnicity (women)	Brahmin and Chhetri	102	19.6	Ethnicity (women)	Brahmin and Chhetri	155	50.2
	Janajatis	155	29.5		Janajatis	66	21.3
	Madhesi	157	30.0		Madhesi	51	16.7
	Dalit	66	12.6		Dalit	37	11.8
	Muslims and others	43	8.3	Managed by	Private	97	31.3
Education (women)	Never been school	155	29.6		Public	212	68.7
	< 10 years	216	41.4	**Intermediary**			
	SLC and above	152	29.0	Province	One	24	7.9
Managed by	Private	73	14.0		Two	46	14.8
	Public	450	86.0		Three	110	35.7
**Intermediary**					Four	38	12.4
Province	One	89	16.9		Five	48	15.6
	Two	154	29.5		Six	19	6.2
	Three	116	22.2		Seven	23	7.3
	Four	25	4.8	Women’s age (years)	15–19	36	11.6
	Five	84	16.0		20–24	130	42.2
	Six	20	3.9		25–29	96	31.1
	Seven	35	6.7		30 and above	47	15.1
Region	Mountain	16	3.0	Companion in delivery	No	93	30.2
	Hill	172	32.9		Yes	216	69.8
	Terai	335	64.1	**Health system**			
Women’s age (years)	15–19	120	23.0	HF capacity	Low	123	39.8
	20–24	230	43.9		Medium	105	34.0
	25–29	118	22.6		High	81	26.1
	30 and above	55	10.5	Supervision of staff	No	119	38.5
Waiting time	Immediately	149	28.4		Yes	190	61.5
	Up to 30 min	251	48.0	Feedback collection	Yes	249	80.6
	> 30 min	123	23.5		No	60	19.4
Facility types	PHCCs and above	303	57.9	QA activities	No	214	69.3
	HPs and clinics	220	42.1		Yes	95	30.7
**Health system**				Timely decision	No	25	8.0
HF capacity	Low	175	33.5		Yes	284	92.0
	Medium	174	33.3	Providers	Nurse and other	203	65.8
	High	174	33.3		Doctor	106	34.2
Supervision of staff	No	172	33.0	PNC mothers	Nurse and others	137	44.5
	Yes	351	67.0		Doctor	172	55.5
HF meeting	No	64	12.3	PNC newborns	Nurse and others	134	43.2
	Sometimes	81	15.5		Doctor	175	56.8
	Monthly	378	72.3	First baby	No	136	44.1
Feedback collection	Yes	326	62.4		Yes	173	55.9
	No	197	37.6	Delivery	Normal	239	77.4
QA activities	No	392	74.9		Assisted	70	22.6
	Yes	131	25.1	Aama program	No	78	25.1
Availability of waiting area	No	39	7.4		Yes	231	74.9
	Yes	484	92.6				
Provider category	GP/Specialists	77	15.2				
	MBBS	23	4.5				
	Nursing	392	77.5				
	Paramedics	14	2.8				
Supervision to staff	No	265	50.5				
	Yes	258	49.5				
Problem felt	No	224	42.9				
	Yes	299	57.1				
Need to pay	Yes	168	32.1				
	No	355	67.9				

### Determinants of utilisation of better technical quality routine MNH services

Table 2 shows the patterns of utilisation of better technical quality ANC services, and delivery and PNC services. Publicly managed HFs (53%), ANC services provided by nursing staff (55%), and those HFs from province seven (72%) were found to have high proportion of utilisation of better technical quality ANC services compared to their reference groups. Women who attended at HFs with better capacity (60%), who belonged to Brahmin/Chhetri ethnic groups (64%) or who had attained 10th grade of education (62%) utilised better quality ANC services. Conversely, women utilised better quality delivery and PNC services in private HFs (75%), Janajati women (59%), women aged 30 years or above (68%), if doctors provided MNH services (60%). However, only 11% of women from province two received better quality delivery and PNC services. Among seven provinces in Nepal, province two is located in the Southern part of the country and has a low literacy rate and densely populated [63], while province six has the lowest status of socioeconomic indicators and the remotest province [64].

**Table 2:** Utilisation of better technical quality of routine MNH services stratified by independent variables in Nepal, 2015

Pregnant women attended HFs for their first ANC visit (***N*** = 523) who received better quality ANC services (%)					Postpartum women discharged from HFs (***N*** = 309) who received better quality delivery and PNC services (%)				
Determinants	Categories	Total frequency	% of better quality	*p*	Determinants	Categories	Total frequency	% of better quality	*p*
**Structural**					**Structural**				
Ethnicity	Brahmin/Chhetri	103	63.8	0.022	Ethnicity	Brahmin/Chhetri	155	57.1	< 0.001
	Janajatis	155	51.9			Janajatis	66	58.9	
	Madhesi	157	45.8			Madhesi	51	23.6	
	Dalit	66	47.9			Dalit	37	40.4	
	Muslims	43	27.1		Managed by	Private	97	74.6	
Education	No schooling	155	36.4	0.001		Public	212	38.7	< 0.001
	Up to 10 grades	216	61.7		**Intermediary**				
	≥SLC	152	46.5		Province	1	24	42.3	< 0.001
Managed by	Private	73	30.7	< 0.001		2	46	11.2	
	Public	450	52.9			3	110	69.0	
**Intermediary**						4	38	46.7	
Province	One	89	43.4	< 0.001		5	48	58.1	
	Two	154	32.9			6	19	55.6	
	Three	116	51.5			7	23	26.6	
	Four	25	69.8		Women’s age (years)	15–19	36	44.5	0.013
	Five	84	66.3			20–24	130	38.9	
	Six	20	65.5			25–29	96	58.1	
	Seven	35	72.1			≥30	47	68.1	
Waiting time	Immediately	149	47.2		Companion in delivery	No	93	55.5	0.32
	Up to 30 min	251	52.2	0.427		Yes	216	47.6	
	> 30 min	123	46.7		**Health system**				
Region	Mountain	16	53.9	0.117	HF capacity	Low	123	59.9	0.160
	Hill	172	57.9			Medium	105	45.4	
	Terai	335	45.5			High	81	40.6	
Women’s age (years)	15–19	120	53.8	0.782	Supervision of staff	No	119	66.3	0.010
	20–24	230	49.7			Yes	190	39.7	
	25–29	118	44.7		HF meeting	Never	47	53.5	0.812
	≥30	55	52.8			Sometimes	50	55.0	
**Health system**						Monthly	211	48.0	
HF capacity	Low	175	40.2	0.036	Feedback	Yes	249	55.2	
	Medium	174	59.7			No	60	28.2	
	High	174	48.6		QA activities	No	214	45.3	0.120
Facility types	PHCCs and hospitals	303	49.1	0.782		Yes	95	60.5	
	HPs and clinics	220	50.8		Aama program	No	78	67.7	0.051
Supervision of staff	No	172	41.4	0.087		Yes	231	44.0	
	Yes	351	54.0		Decision to seek care	Late	25	60.3	0.403
HF meeting	Never	64	50.9	0.961		Timely	284	49.0	
	Sometimes	81	47.8		Providers	Nurse	203	43.1	0.006
	Monthly	378	50.1			Doctors	106	63.1	
Feedback	Yes	326	52.2	0.320					
	No	197	45.9						
QA activities	No	392	50.0	0.921	PNC mothers	Nurses	137	37.8	0.006
	Yes	131	49.3			Doctor	172	59.7	
Waiting area	No	39	38.2	0.309	PNC-Newborn	Nurses	134	35.7	0.003
	Yes	484	50.7			Doctor	175	60.8	
HW category	GP/Specialists	77	29.5	0.004	First baby	No	136	51.8	0.576
	MBBS	23	26.3			Yes	173	48.5	
	Nursing	392	55.0		Delivery	Normal	239	46.4	0.047
	Paramedics	14	31.5			Assisted	70	62.0	
Supervision of staff	No	265	45.8	0.209					
	Yes	258	54.0						
Problem felt	No	224	56.1	0.067					
	Yes	299	45.1						
Need to pay	Yes	168	43.7	0.135					
	No	355	52.7						

Note: *p*-values based on Fisher exact test

### Determinants of utilisation of better technical quality MNH services

Table 3 shows the logistic regression analysis of the utilisation of better quality MNH services. Out of the 18 independent variables examined for the utilisation of quality ANC services in bivariable regression analysis, seven variables including structural (women’s ethnicity and education, management authority), intermediary (province, region), health system (HF capacity, types of health workers) were significantly positively associated with better quality ANC services. Out of 17 variables examined in the bivariable regression analysis, nine variables, which included structural (ethnicity, types of management), intermediary (province), health system (external supervision, feedback collection, types of providers, providers of PNC-mother, types of providers of PNC-newborn, mode delivery) were significantly associated with utilisation of better quality of delivery and PNC services.

**Table 3:** Binomial logistic regression for the determinants of utilisation of better technical quality of routine MNH services in Nepal, 2015

Pregnant women attended HFs for their first ANC visit (N = 523)				Postpartum women discharged from HFs (N = 309)			
Determinants	Categories	cOR (95% CI)	aOR (95% CI)	Determinants	Categories	cOR (95% CI)	aOR (95%CI)
**Structural**				**Structural**			
Ethnicity (women)	Brahmin/Chhetri	1.00		Ethnicity (women)	Brahmin/Chhetri	1.00	
	Janajatis	0.46(0.22,0.94) *			Janajatis	1.07(0.57, 2.03)	
	Madhesi	0.33(0.16,0.69) **			Madhesi	0.23(0.10, 0.51) ***	
	Dalit	0.43(0.20,0.94) *			Dalit	0.51(0.21, 1.26)	
	Muslims	0.20(0.08,0.53) **		Managed by	Public	1.00	1.00
Education	No schooling	1.00			Private	4.64(2.05, 10.48) ***	2.63(1.14, 6.08) *
	Up to 10 grades	2.21(1.20,4.08) *		**Intermediary**			
	SLC and above	1.86(0.99,3.50)		Province	One	1.00	1.00
Managed by	Public	1.00			Two	0.17(0.04, 0.67) *	0.15(0.03, 0.63) *
	Private	0.50(0.28,0.87) *			Three	3.03(0.83,11.08)	2.04(0.57, 7.31)
**Intermediary**					Four	1.19(0.29, 4.85)	0.94(0.21, 4.21)
Province	One	1.00	1.00		Five	1.89(0.46, 7.70)	1.58(0.36, 7.00)
	Two	0.72(0.26,1.94)	0.52(0.19,1.38)		Six	1.71(0.47, 6.20)	2.94(0.68, 12.69)
	Three	1.82(0.72,4.60)	2.11(0.84, 5.32)		Seven	0.49(0.13, 1.91)	0.59(0.15, 2.33)
	Four	4.55(1.58,13.08) **	4.03(1.56, 10.40) **	Women’s age (years)	15–19	1.00	
	Five	2.32(0.88,6.08)	1.60(0.63, 4.04)		20–24	0.80(0.39, 1.61)	
	Six	4.01(1.14,14.11) *	3.28(0.90,12.01)		25–29	1.73(0.78, 3.86)	
	Seven	3.88(1.43,10.49) **	2.77(0.94, 8.16)		≥30	2.67(0.96, 7.38)	
Waiting time	Immediately	1.00		Delivery Companion	No	1.00	
	Up to 30 min	1.22(0.70,2.10)			Yes	0.73(0.39, 1.37)	
	> 30 min	0.98(0.50,1.92)					
Region	Terai	1.00		**Health system**			
	Mountain	1.95(0.48,7.89)		HF capacity	Low	1.00	
	Hill	1.84(1.08, 3.15) *			Medium	0.56(0.23, 1.32)	
Women’s age (years)	15–19	1.00			High	0.46(0.20, 1.05)	
	20–24	1.42(0.69,2.90)		Supervision of staff	No	1.00	
	25–29	1.13(0.58,2.21)			Yes	0.33(0.14, 0.78) *	
	≥30	1.14(0.44,2.99)		HF Meeting	Never	1.00	
Facility types	PHCCs and hospitals	1.00			Sometimes	1.06 (0.20, 5.72)	
	HPs and clinics	0.78(0.43, 1.39)			Monthly	0.80(0.35, 1.85)	
**Health system**				Feedback collection	Yes	1.00	
HF capacity	Low	1.00	1.00		No	0.32(0.16, 0.64) **	
	Medium	2.21 (1.07, 4.56) *	2.12(1.03, 4.35) *	QA activities	No	1.00	
	High	1.41(0.67, 2.97)	1.27(0.55, 2.94)		Yes	1.85(0.85, 4.02)	
Supervision of staff	No	1.00		Aama program	No	1.00	
	Yes	1.79(0.91,3.52)			Yes	0.37(0.14, 1.02)	
HF meeting	Never	1.00		Decision	No	1.00	
	Sometimes	0.74(0.22,2.47)			Timely	0.63(0.21, 1.89)	
	Monthly	1.04(0.43,2.53)		Providers	Nurse	1.00	
Feedback collection	Yes	1.00			Doctors	2.26(1.27, 4.04) **	
	No	0.75(0.41,1.37)					
Quality assurance	No	1.00					
	Yes	1.04(0.55,1.97)		PNC Mothers	Nurses	1.00	
Waiting area	No	1.00			Doctor	2.44(1.29, 4.62) **	
	Yes	1.39(0.53,3.66)		PNC Newborn	Nurses	1.00	1.00
HWs category	GP/Specialists	1.00	1.00		Doctor	2.79(1.44, 5.42) **	2.14(1.13, 4.04) *
	MBBS	0.85(0.18,4.07)	1.00 (0.23, 4.35)	First baby	No	1.00	
	Nursing	2.06(1.07,3.94) *	2.89(1.33, 6.29) **		Yes	0.88 (0.55, 1.40)	
	Paramedics	0.91(0.21,3.96)	0.89(0.21, 3.74)	Delivery	Normal	1.00	
Staff supervision	No	1.00	1.00		Assisted	1.88(1.00, 3.52) *	
	Yes	1.71(0.96,3.03)	1.71(1.01,2.92) *				
Problem felt (clients)	No	1.00					
	Yes	0.70(0.42,1.14)					
Need to pay	Yes	1.00					
	No	1.19 (0.71,1.99)					

Significance at ** p < 0.01, * p < 0.05. Variables which had p < 0.2 included in the final model for each outcome variable. For each outcome variable, independent binomial logistic regression analysis was consudcted adjusting for covariates listed in the respective column. Goodness of fit test (Hosmer Lemeshow test) for utilisation of technical quality for ANC services (p = 0.896). Goodness of fit test (Hosmer Lemeshow test) for utilisation of technical quality of delivery and PNC services (p = 0.793). These figures show that our models are the best fit.

In the multivariable logistic regression analyses, four variables (HF capacity, province, types of health providers, and staff’s supervision) were significantly positively associated with utilising better-quality ANC services. Compared to women from province one, women living in province four (adjusted odds ratio (aOR) = 4.03; 95% CI: 1.56, 10.40) received better quality ANC services. Province four has a comparatively high number of HFs and a better road network to reach HFs [64], and it is a developed province with a higher human development index compared to other provinces. Women utilised better quality (aOR = 2.89; 95% CI: 1.33, 6.29) ANC services if they received services from the nursing staff compared to physicians. If pregnant women received ANC services from health providers who received specific supervision in the past 4 months, the odds were higher (aOR = 1.71; 95% CI: 1.01, 2.92) for better quality ANC services compared to their reference category. Three variables (management authority, province, providers of PNC-newborn) were significantly associated with utilisation of better quality of delivery and PNC services after adjusting all covariates at *p* < 0.05. The odds were more than two times higher for the utilisation of better technical quality delivery and PNC services (aOR = 2.63; 95% CI: 1.14, 6.08) if women received services at private HFs. Women from province two had 85% lower odds of the utilisation of better technical quality delivery and PNC services (aOR = 0.15; 95% CI: 0.03, 0.63) (Table 3).

## Discussion

This study demonstrated that women utilised better quality care from private than public HFs. In Nepal, the number of private HFs has increased since 1995, but basic health services, including MNH services, are not freely available in private HFs. However, women utilised poor quality delivery and PNC services in HFs of province two. Compared to other provinces, province two lies in the Southern part of the country, bordered with north India, has better access and transportation systems. In addition, women in this province generally have low literacy and awareness of health information on their health needs, and availability of health services in HFs [63]. Health staff supervised by a higher authority provided better quality MNH services. Supervision and monitoring of HFs and staff at a lower level can improve the management and capacity for better quality services delivery [65]. Further, women utilised better technical quality of MNH services from the nursing staff than physicians’ services. In Nepal, trained SBAs, usually auxiliary nurse midwives (all are females) provide routine MNH services, thus, MNH services provided by nursing cadre can be argued as of better technical quality.

Women utilised better technical quality of quality ANC and institutional delivery in private HFs. In Nepal, while the share of private HFs in urban areas is increasing, private health services, including routine MNH services, are expensive due to high OOP expenditure and lack of financial risk protection in health care [34, 66], may charge up to 15 USD per consultation [67]. Such a high cost of care could limit access to quality health services, especially for disadvantaged women and exacerbate health inequities. Women’s access to MNH services in private HFs can be improved through the scaling up of the Safe Delivery Incentive Program (SDIP) in additional private HFs. In addition, the NHIP can be linked with routine private MNH services through which OOP expenses can be reimbursed [36]. Furthermore, women should receive quality health services irrespective of the types of HFs management (private or public). Adopting a public-private partnership (PPP) model could increase disadvantaged population’s use of quality MNH services in private sector HFs. Concurrently, the quality of care in public sector HFs needs to be improved as well for better delivering quality care for MNH outcomes. Improving the quality of care in public HFs is particularly important in Nepal as peripheral and public HFs provide more than 80% of maternity services [32].

In this study, level of education and ethnicity were not associated with utilisation of quality MNH services in the final regression model despite being significantly associated in bivariable analysis. Further analysis of household survey data suggests that women of disadvantaged ethnicity or low level of education have poor access to quality ANC services in Nepal [15], and low effective coverage of MNH services [17]. Usually, in Nepal, women of privileged groups prefer to receive MNH services at private HFs [66]. Further studies could confirm the level of quality of care utilised by women with educational and ethnic disadvantages in Nepal.

This study showed women utilised better quality ANC services if they attended HFs with better health system inputs (structural quality). This underscores the need for building HF capacity through system inputs, including trained health workers, equipment, medicine, supplies and protocols/guideline, infrastructure. Health system readiness/inputs are hindered by several supply-side barriers, including poor health system readiness and system governance, lack of health workforce accountability [68, 69], including lack of adequate trained staff [70]. Therefore, program and policy efforts should focus on improved management and health governance systems for better HF capacity for quality MNH services.

Women received poor quality MNH services in HFs of province two, where the highest number of childbirths occur annually [32]. Therefore, improving the quality of HFs in province two should be a priority in addressing inequities in access to quality care and health outcomes. A previous study of care for NCDs also found HFs of province two had poor health system readiness [25]. In addition, studies also suggest women’s access to reproductive, maternal and child health issues need to be improved [32, 63]. Women in the province two [37], especially those who are more disadvantaged, also experience some demand and supply-side barriers in accessing quality MNH services [32, 63], including lack of trained SBAs in HFs, inadequate supply of medicine, equipment and lack of necessary infrastructure for better HF capacity [26]. Demand-side barriers include poor access to information and awareness, language barriers, ethnic disadvantage, and sociocultural barriers such as shyness, especially with male health care providers [68, 71]. Thus, the provincial and local governments of this province could recruit trained staff from the local municipalities who can understand local context, languages, culture, and address context-specific issues.

The current study identified if staff were supervised by a higher authority in the last 4 months, women utilised better technical quality MNH services in HFs. Studies suggest staff supervision can improve quality MNH services: by improving management functions and improving technical capacity for the delivery of clinical services [72, 73]. Supervision and monitoring of HFs and HWs at the peripheral HFs could improve the quality of health services in Nepal [65]. In Tanzania, a study reported that supervision visits improved health management functions in peripheral HFs and delivered better quality primary health care (PHC) services [74]. Therefore, regular supervision and monitoring, onsite coaching of health workers at lower-level HFs are vital to improving the utilisation of better quality MNH services in Nepal.

Finally, while in Nepal, trained nurse-midwives provide much of the routine MNH services, especially normal deliveries and immediate newborn care services for normal neonates. The current study identified that women who received care from nursing staff also received better quality services. In Nepal, nursing staff are females only (since 2020, males are also allowed to study nursing program). Culturally, Nepali women feel more comfortable sharing reproductive health needs with the nursing cadre, especially reproductive and maternal newborn and child health services [75]. Evidence suggests women across South Asia prefer nursing staff and female physicians compared to male health providers [72, 76]. In the context of the federal health system governance of Nepal, local governments have the funds and authority for design and implementation of context-specific health programs [30], the local recruitment of nurse-midwives could be possible in the peripheral HFs. Though current study identified that nurse-midwives provided better routine MNH services for normal deliveries and routine newborn care, but are often poorly skilled in handling vulnerable newborns (e.g., sick newborns, premature babies) [77]. Therefore, nursing staffs need further training and support, especially with newborns requiring higher levels of care, such as low birth weight babies, preterm, or babies with a breathing problem. As doctors are usually not available at the lower level of HFs, transferring skills to nurse-midwives who work at the lower level is critical.

### Implications for policy and programs

This study has some policy and programmatic implications. Firstly, this study suggests improving the quality of care in public facilities. Secondly, context-specific interventions need to implement at peripheral HFs; provision of local health staff, including nurse-midwives, could improve the delivery and utilisation of quality MNH services. Importantly, provincial and local governments should focus on the strengthening health system for better health service readiness, delivery and utilisation of quality MNH services. Thirdly, peripheral HFs and health workers should receive monitoring and onsite coaching for improved quality of care for women/newborns with disadvantaged groups.

### Strengths and limitations of the study

This study has some strengths and limitations. First, this study has analysed data from nationally representative health facility survey with multiple data sources. Secondly, this study created the composite measure of quality assessment and determinants of its utilisation. This study has some limitations, firstly, findings of this study do not predict the causality rather shows the correlation. Secondly, some of the categories of the independent variable (e.g., province) have a small sample size (e.g., province six, 19 postpartum women), there is likely to be a large degree of random error. Categories with small sample sizes should be interpreted with caution due to the inadequate precision, and the magnitude of these effect estimates need to be confirmed in future studies with larger sample sizes. Thirdly, this study represents the status of quality of care at the time of data collection in 2015 [29] as there has not been a recent nationwide survey and available data to assess the utilisation of quality MNH services. Finally, this study represents the quality of health care utilisation 5 years ago; as the country has changed from its unitary governance to federal governance, the situation may have changed after the implementation of the federal health system.

## Conclusions

Women utilised better quality MNH services in private HFs; nursing staff provided better quality MNH services. Several approaches can be implemented to improve the utilisation of better technical quality of MNH services. Access to ANC services in privately managed HFs could be improved through the implementation of the public- private-partnership (PPP) strategy such as the demand-side financial program (e.g., SDIP). Routine MNH services can be made available free of cost at private sector HFs if the National Safe Motherhood Program is linked with the NHIP. Provincial and local governments require to strengthen the local/municipal health systems to designand implement context-specific interventions such as trained local SBAs, onsite coaching of health staff, supervision, and monitoring of HFs, especially usually visit peripheral HFs.

## Supplementary Information

**Additional file 1 **: **Table S1.** Description of independent variables included in the utilisation of better quality MNH services in Nepal. **Table S2.** Items included in the HF capacity assessment for antenatal care services. **Table S3.** Items included in the HF capacity assessment for delivery and postnatal services. **Table S4.** Observations and uptake of ANC interventions for the measurement of the technical quality of ANC services. **Table S5.** Observations and uptake of delivery and PNC interventions for the measurement of the technical quality of delivery and PNC services.

## Data Availability

Data used in this study are publicly available secondary data obtained from the DHS **(**https://dhsprogram.com/data/available-datasets.cfm**)** program.

## Abbreviations

ANC: Antenatal Care
LMICs: Low and lower-middle-income countries
MNH: Maternal and newborn health
PNC: Postnatal Care
SDIP: Safe Delivery Incentive Program
SPA: Service Provision Assessment
HF: Health Facility
SARA: Service Availability and Readiness Assessment
SDGs: Sustainable Development Goals
HFs: Health facilities
QA: Quality assurance
PHCC: Primary health care centres
HP: Health post
PPP: Public-private-partnership
UHC: Univesal Health Coverage
