# Supplementary material for: Utilisation of quality antenatal, delivery and postnatal care services in Nepal: An analysis of Service Provision Assessment
Source: Global Health. 2021 Sep 6;17:102. doi: 10.1186/s12992-021-00752-x (PMC8419903; doi:10.1186/s12992-021-00752-x)
Supplement: Supplementary file 1 — Additional file 1 : Table S1. Description of independent variables included in the utilisation of better quality MNH services in Nepal. Table S2. Items included in the HF capacity assessment for antenatal care services. Table S3. Items included in the HF capacity assessment for delivery and postnatal services. Table S4. Observations and uptake of ANC interventions for the measurement of the technical quality of ANC services. Table S5. Observations and uptake of delivery and PNC interventions for the measurement of the technical quality of delivery and PNC services. [file 12992_2021_752_MOESM1_ESM.doc]

## Supplementary file 1

Table S1: Description of independent variables included in utilisation of better technical quality MNH services in Nepal (Questionnaire NHFS/SPA original report annex C, section (HF inventory), health workers interview (page 317), observation of ANC consultation (page -333), and exit interview with postpartum women (page 403)).

| **Variables** | **Categories** | **Descriptions** |
| --- | --- | --- |
| Independent variables |  |  |
| **Structural** |  |  |
| Ethnicity of women | Brahmin/Chhetri, Janajatis, Terai/Madhesi other castes, Dalits | Brahmin/Chhetri is privileged ethnicity in Nepal |
| Managing authority | Private, Public | Management of HFs |
| Education (women) | Never been school, <10 years of schooling,  SLC and above | The education level of women |
| **Intermediary** |  |  |
| Province | 1,2,3,4,5,6, 7 | Location of HFs |
| Region | Mountain, Hill, Terai | Region of HFs |
| Age of women (years) | 15-19,  20-14,  25-29,  30 and above | Age of women (pregnant women, and postpartum women) |
| Facility type | Primary health care center (PHCCs) and above,  Peripheral HFs | Health posts and below HFs which do not have a bed for admission |
| Companion at HFs | No; Yes | Companion during delivery (especially husband, mother-in-law or other family members) |
| Waiting time | >30 minutes; Immediately or  <30 minutes | Waiting time to see the providers in health facilities |
| **Health system** |  |  |
| HF capacity | High,  Medium, and  Low | Calculated using principal component analysis taking several items listed in tables 2 and 3 specific ANC serivces, or deliveyr and PNC services |
| Checked by | Nurse and other; Doctor | Checked before discharge |
| Mode of delivery | Normal delivery; Instrumental delivery | Instrumental delivery includes forceps and c-section delivery |
| The problem felt at the HFs | No; Yes (felt) |  |
| Aama program (or Safe Delivery Incentive Program) | No; Yes | Under the Aama program, women get money if they gave birth at HFs. Aama program in Nepali translation of SDIP. |
| Need to pay by clients | No; Yes | Monitory payment for services |
| External supervision of HFs | No; Yes | Supervision of HFs in the last four months |
| HF management meeting | Never/no, Sometimes, Regularly/monthly | Frequency of facility management meeting |
| System for collecting opinion | No; Yes | Feedback collection of the service of HFs |
| Quality assurance in the past year | No; Yes | Quality assurance mechanism in HFs |
| Protected client waiting area | No; Yes | Client waiting areas available in HFs |
| Qualification of providers | Specialist,  MBBS,  Nursing,  Paramedics | Educational qualification of providers |
| Supervision in the last four months (providers) | No; Yes | Supervision of health providers in the last four months |
| Providers' type | Nurse and other; Doctor | Categories of health services providers |
| **Outcome variables** |  |  |
| Utilisation of technical quality ANC services | Poor quality =0;  better quality =1 | Identified based on data of observation of health services delivery, and client exit interviews with pregnant women who attended for first ANC visit on the day of survey |
| Utilisation of technical quality delivery and PNC services | Poor quality =0;  better quality =1 | Identified based on data of observation of health services delivery, and client exit interviews with postpartum mothers who discharged on the day of survey |
| SLC: School leaving certificate; HFs: Health facility | | |

Table S2: Items included in the assessment of HF capacity (structural quality of HFs) for ANC services Nepal (Questionnaire NHFS/SPA original report annex C section 14, pages 280-283).

| **Status of general readiness** | **Yes % (N=269)** | **Availability of equipment** | **Yes % (N=269)** |
| --- | --- | --- | --- |
| 24-hour staff availability | 54.9 | Water for infection prevention | 68.7 |
| Water supply | 92.4 | Soap for Infection Prevention (IP) | 72.5 |
| Client latrine | 94.1 | Disinfectant for IP | 66.8 |
| Emergency transport | 82.3 | Autoclave services | 98.7 |
| Client waiting area | 94.2 | Digital blood pressure (BP)apparatus | 3.0 |
| Landline phone | 55.2 | BP set manual | 95.7 |
| Electricity service | 88.1 | Stethoscope | 97.6 |
| **Availability of ANC services** | **Yes % (N=269)** | Examination light | 56.1 |
| Iron tablet distribution | 74.5 | Fetoscope available | 95.7 |
| Folic acid distribution | 63.4 | Weighing scale | 98.9 |
| Tetanus toxoid service | 94.1 | Exam table | 98.3 |
| Albendazole tablets distribution | 96.3 | Tape fundal height | 44.0 |
| Misoprostol distribution | 23.1 | Thermometer | 78.6 |
| Urine test service | 35.4 | **Availability of medicine** | **Yes % (N=269)** |
| Haemoglobin test services | 31.9 | Misoprostol tablets | 36.4 |
| ANC counselling | 99.6 | Iron-folic tabs | 71.8 |
| Birth preparedness package counselling | 99.0 | Tetanus toxoid vaccine | 43.0 |
| Family Planning counselling | 95.8 | Albendazole tablets | 84.8 |
| Counselling on prevention of HIV/AIDS | 94.9 | **Staff training and guidelines** | **Yes % (N=269)** |
| Breastfeeding counselling | 95.9 | ANC guideline | 27.0 |
| Newborn care counselling | 95.2 | IEC materials for ANC | 75.3 |
| PNC counselling | 96.0 | Supervision | 18.6 |
| Weighting clients | 92.0 | ANC screening | 18.6 |
| Measure BP services | 93.1 | ANC counselling | 20.9 |
| Health education service | 21.0 | Complication and management | 20.9 |
| Urine portion test | 44.8 | Nutritional assessment | 13.0 |
| Anaemia test services | 53.4 | Other training | 4.1 |
| HIV test and counselling | 55.9 |  |  |
| Measurement of height | 34.7 |  |  |

Table S3: Items included in the assessment of HF capacity (structural quality of HFs) for delivery and PNC services (Questionnaire NHFS/SPA original report annexe C section 16, pages 287-292).

| **Availability of equipment** | **Yes, % (N=109)** | **Status of general readiness** | **Yes, % (N=109)** | **Availability of medicines** | **Yes, % (N=109)** |
| --- | --- | --- | --- | --- | --- |
| Heat source available | 82.2 | Landline phone | 93.0 | Injectable antibiotic | 84.2 |
| Examination light | 96.1 | Mobile phone | 26.7 | Tablet oxytocin | 99.2 |
| Dee-lee suction | 56.8 | Water supply | 96.1 | Tablet magnesium sulphate | 91.4 |
| Bag and Mask | 98.4 | Client latrine | 96.1 | Intravenous fluid | 98.4 |
| Thermometer | 92.1 | Protected client waiting area | 96.1 | Betadine solution | 96.8 |
| Infant scale | 99.2 | Electricity service | 96.9 | Chlorhexidine tube | 47.3 |
| Fetescope | 95.9 | Emergency transport | 94.6 | Calcium gluconate | 76.0 |
| Blood pressure set | 96.8 | 24 Hr duty call for delivery services | 80.2 | Nifedipine capsule | 62.0 |
| Stethoscope | 98.4 | **Staff, guideline, and training** | **Yes, % (N=109)** | **Availability of newborn services** | **Yes, % (N=109)** |
| Delivery bed | 99.2 | IMPAC training | 40.3 | Newborn resuscitation | 87.0 |
| Delivery set | 97.6 | Routine labor and delivery | 34.8 | Kangaroo mother care | 85.7 |
| Cord clamper | 92.9 | AMTSOL | 32.7 | Injectable antibiotic available | 84.2 |
| Vaginal speculum | 94.4 | MNH update emergency obstetric care | 28.0 | Skin to skin contact | 88.5 |
| Cord cutting blade | 96.1 | Neonatal resuscitation training | 33.7 | Wrapping baby | 100 |
| Epitomy set | 96.1 | Exclusive breastfeeding training | 28.9 | Immediate breastfeeding | 98.4 |
| Suturing blade | 98.3 | Neonatal sepsis management | 21.8 | Head to toe | 98.4 |
| Needle holder | 96.8 | Thermal care | 24.2 | Weighing newborn | 100 |
| Forceps | 91.3 | Cord cutting training | 28 | Use of chlorhexidine | 53.6 |
| Sponge holder | 96.8 | Kangaroo Mother Care training | 31.2 | Delayed bathing | 70.5 |
| Blank paratograph | 82.2 | Other training | 2.4 | **Availability of delivery services** | **Yes, % (N=109)** |
| Baby wrappers four sets | 86.5 | Supervision Health worker | 97.7 | Antibiotics parental | 91.4 |
| Nayano Jhola set | 40.9 | External supervision in the last four months | 70.5 | Oxytocin parental | 98.4 |
| Water available in the delivery room | 95.3 | Reproductive health guideline | 27.7 | Anticonvulsant parental | 52.1 |
| Soap available in the maternity room | 94.5 |  |  | Injectable antibiotic | 84.2 |
| Alcohol for hand rub in the maternity room | 45.6 |  |  | Use of paratograph | 85.4 |
| Latex gloves | 97.6 |  |  |  |  |
| Disinfectant available in the maternity room | 92.9 |  |  |  |  |
| Autoclave services | 100 |  |  |  |  |

Note IMAC: Skilled birth attendant, Integrated management of pregnacy and childbirth. AMTSOL: Acute management of the third stage of labour.

Table S4: Utlisation of ANC interventions (items used for the measurement of the technical quality of ANC services) obtained from the observation of the interaction of pregnant women and providers and exit interviews with pregnant women who visited HFs for first ANC visit in SPA 2015. See for the tool (SPA original report observation of ANC consultation and exist interview (page-333)).

| **ANC interventions** | **Yes (N=523), %** |
| --- | --- |
| Asked clients age | 53.6 |
| Mensuration date asked | 25.4 |
| The previous history asked | 100 |
| Asked veginal bleeding | 100 |
| Asked for eye vision | 100 |
| Checked swelling | 100 |
| Foetal measurement | 100 |
| Physical examination | 100 |
| Measured Blood Pressure | 100 |
| Weight took | 85.3 |
| Palm checked | 62.1 |
| Checked for oedema | 42.9 |
| Palpated abdomen | 36.0 |
| Checked breast | 22.5 |
| Listen to foetal heartbeat | 83.6 |
| Tested haemoglobin | 50.2 |
| Tested blood grouping | 47.9 |
| Counselling on urine test | 100 |
| Counselling on syphilis test | 25.2 |
| Counselling on HIV test | 100 |
| Counselling health pregnancy | 24.3 |
| Nutritional counselling | 52.9 |
| Counseling on iron prophylaxis | 100 |
| Counselling on deworming | 49.8 |
| Tetanus Toxoid counselling | 42.6 |
| Institutional delivery promotion | 100 |
| Newborn postpartum recommendation | 100 |
| Advise dangers signs during pregnancy | 100 |
| Advise dangers signs during delivery | 100 |
| Advise dangers signs of newborns | 100 |
| Advise danger signs during postpartum | 100 |
| Received iron | 56.2 |
| Nutritional counselling | 52.4 |
| Pregnancy complication counselling | 22.7 |
| Advise what to do after complication | 100 |
| Advise preparation for delivery | 9.6 |
| Plan for delivery | 17.2 |
| Counselling for exclusive breastfeeding (EBF) | 8.1 |
| Family planning counselling | 4.2 |
| Advise EBF within one hour | 6.3 |

Table S5: Utlisaiton of delivery and PNC interventions (items used for the measurement of the technical quality of delivery and PNC services) obtained from client exit interview with postpartum women who discharged on the day of HFs survey in SPA 2015. See for the tool (SPA original report: exit interview with postpartum women (page 403)).

| **Delivery and postnatal interventions** | **Yes (N=309), %** |
| --- | --- |
| Measured Blood Pressure | 81.0 |
| Checked pulse | 72.9 |
| Checked temperature | 57.3 |
| Checked swelling | 43.3 |
| Checked perineum | 61.5 |
| Checked breast | 43.3 |
| Asked for urine | 63.4 |
| Checked uterine | 56.7 |
| Asked if any bleeding | 67.3 |
| Cord care | 54.4 |
| Breast Feeding advise | 82.6 |
| Family Planning advise | 25.1 |
| PNC advise | 63.3 |
| Would situation examination | 49.0 |
| Advise on dangers signs | 38.2 |
| Checked baby's temperature | 67.6 |
| Checked baby's breathing status | 64.4 |
| Checked colour and movement | 58.4 |
| Cord examination | 58.9 |
| Checked skin | 32.0 |
| Checked eyes | 36.2 |
| Checked for Jaundice | 47.8 |
| Asked on breastfeeding situation | 84.7 |
| Asked on immunisation situation | 64.8 |
